# Association of Body Mass Index (BMI) With Breath-Holding Time and Six-Minute Walk Distance in Apparently Healthy Adults

**DOI:** 10.7759/cureus.91777

**Published:** 2025-09-07

**Authors:** Himani H More, Sujata Khatal

**Affiliations:** 1 Physiology, Autonomous State Medical College Amethi, Amethi, IND; 2 Pharmacy, Amrutvahini Institute of Pharmacy, Sangamner, IND

**Keywords:** body mass index: bmi, breath holding time, obesity, overweight, six minute walk distance

## Abstract

Introduction: Body mass index (BMI) is a simple anthropometric index widely used to classify individuals based on weight status. Alterations in BMI may influence respiratory function and exercise capacity. This study aimed to assess the association of BMI with breath-holding time (BHT) and six-minute walk distance (6MWD) in apparently healthy adults. Methods: A cross-sectional observational study was conducted in the Department of Physiology, King George’s Medical University, Lucknow, after obtaining Institutional Ethics Committee approval. A total of 151 apparently healthy adults aged 20-40 years were recruited and categorized as normal (n=58), overweight (n=85), or obese (n=7) based on WHO BMI criteria. BHT was measured using standard inspiratory breath-hold methodology with nose clips, repeated thrice with a 10-minute interval, and averaged. The 6MWD was assessed on a 30-meter plain ground track marked at 1-meter intervals. Data were analyzed using one-way ANOVA. Results: Mean BHT was highest in the normal BMI group (44.29 ± 12.22 sec), slightly lower in the overweight group (43.68 ± 7.56 sec), and lowest in the obese group (32.43 ± 10.08 sec), with a statistically significant difference (F=4.709, p=0.010). Mean 6MWD was 608.17 ± 77.95 meters in the normal group, 588.31 ± 74.72 meters in the overweight group, and 542.86 ± 81.23 meters in the obese group. The difference in 6MWD was not statistically significant (F=2.829, p=0.062). Conclusion: Higher BMI, particularly obesity, is associated with reduced BHT and a trend towards lower 6MWD, suggesting compromised respiratory endurance and functional exercise capacity even in apparently healthy adults.

## Introduction

Body mass index (BMI) is a universally applied index for classifying underweight, normal weight, overweight, and obesity, as defined by the World Health Organization (WHO) [1]. In India, the prevalence of overweight and obesity has risen significantly in recent decades, contributing to a growing burden of non-communicable diseases [2]. Elevated BMI has been associated with alterations in pulmonary mechanics, reduced respiratory compliance, and increased work of breathing [3]. Furthermore, obesity may impair functional exercise capacity due to excess body mass and cardiovascular strain [4]. Breath holding time (BHT) is a simple, non-invasive, inexpensive and recently validated test [5]. The duration of a voluntary breath-holding during a breath-holding test indirectly reflects the sensitivity of peripheral chemoreflex to carbon dioxide in subjects without cardiac and respiratory diseases [6]. The six-minute walk test (6MWT) is a submaximal exercise test recommended by the American Thoracic Society to assess functional exercise capacity [7]. Both measures have clinical and research utility in evaluating cardiopulmonary function. While the relationship between BMI and pulmonary function tests has been extensively explored, fewer studies have examined its association with BHT and 6MWT in apparently healthy young adults. Understanding this relationship could highlight early functional impairments before overt clinical disease develops. This study aimed to assess the association of BMI with BHT and 6MWD in apparently healthy adults.

## Materials and methods

Study design

This cross-sectional observational study was conducted in the Department of Physiology, King George’s Medical University (KGMU), Lucknow.

Ethics statement

Data collection for the study started after obtaining approval from the Institutional Ethics Committee, letter number 1022/Ethics/2021 with reference code IV PGTS-IIA/P22. The Institutional Review Board (IRB) approval was obtained for the study titled "A Pilot Study on Correlation Between Breath Holding Time Test and 6 Minute Walk Test in Apparently Healthy Individuals". The present study is based on an analysis of the same dataset. While the original approval covered the entire data collection for the thesis, this paper focuses on a specific subset and research question derived from that data which falls within the scope of the originally approved study.

Sample size

The sample size was calculated using the formula n = Z^2p(1-p)/d^2, assuming a 95% confidence level (Z = 1.96), expected proportion p = 0.5 (for maximum variability), and allowable error d = 0.08. This yielded a sample size of approximately 151 [6]. The present study included 151 participants (~50 per BMI group), which aligns with established recommendations for pilot/feasibility studies suggesting 30-75 participants per group to ensure adequate precision of estimates and reliable subgroup comparisons [7,8]. This sample size was feasible within the scope of the original approved dataset while maximizing statistical reliability.

Study population

A total of 151 apparently healthy adults aged 20-40 years were included. Inclusion criteria were apparently healthy individuals aged 20-40 years. Individuals taking any medication, smokers, and those outside the age range were excluded. Participants were divided into three groups according to the WHO BMI classification: normal weight individuals with a BMI in the range of 18.5-24.9 kg/m2, overweight individuals with a BMI range of 25.0-29.9 kg/m2, and obese individuals with a BMI of more than 30 kg/m2.

Study measures

Anthropometric Measurements

Height and weight were measured using standard protocols. BMI was calculated as weight (kg) divided by height squared (m²).

Breath-Holding Time Measurement

Participants were instructed to take a deep inspiration up to their inspiratory reserve volume and hold their breath. Nose clips were applied to prevent nasal breathing. Subjects kept their mouth closed and avoided swallowing. An observer placed one hand on the participant’s diaphragmatic area to detect any expiration. When participants could no longer hold their breath, they signaled by pointing a finger, and the nose clip was removed. The time from the start of inhalation to the initiation of expiration was recorded with a stopwatch. Immediately afterward, oxygen saturation, blood pressure, and pulse rate were measured. The procedure was repeated three times with a 10-minute rest between attempts, and the mean was recorded [9].

Six-Minute Walk Test

The 6MWT was conducted on a 30-meter-long, straight, plain ground marked at every 1 meter. They were wearing comfortable footwear and clothing. Participants walked at their own pace for six minutes, aiming to cover the maximum distance possible. The total distance walked was recorded in meters [10].

Statistical analysis

Data were analyzed using SPSS version 26 (IBM Corp., Armonk, NY, USA). Results are presented as mean ± standard deviation (SD). One-way ANOVA was used to compare groups. A p-value <0.05 was considered statistically significant.

## Results

The mean breath-holding time was highest in the normal weight group (44.29 ± 12.22 seconds), slightly lower in the overweight group (43.68 ± 7.56 seconds), and lowest in the obese group (32.43 ± 10.08 seconds). The ANOVA test revealed a statistically significant difference in BHT across BMI categories (F = 4.709, p = 0.010), indicating that breath-holding time decreases with increasing BMI. The normal weight group covered the greatest distance on average (608.17 ± 77.95 meters), followed by the overweight group (588.31 ± 74.72 meters), and the obese group (542.86 ± 81.23 meters). Although the trend suggests reduced walking distance with higher BMI, the differences were not statistically significant (F = 2.829, p = 0.062). Furthermore, in all three BMI categories - normal weight, overweight, and obese - the 6MWD showed a positive correlation with breath-holding time, indicating that individuals with better respiratory capacity tended to perform better on the functional walking test (Table 1).

**Table 1:** Association of BMI with breath-holding time (BHT) and six-minute walk distance (6MWD).

BMI Category	N	BHT (sec) Mean ± SD	6MWD (m) Mean ± SD
Normal	58	44.29 ± 12.22	608.17 ± 77.95
Overweight	85	43.68 ± 7.56	588.31 ± 74.72
Obese	7	32.43 ± 10.08	542.86 ± 81.23
ANOVA	151	F = 4.709, p = 0.010	F = 2.829, p = 0.062

**Figure 1:**
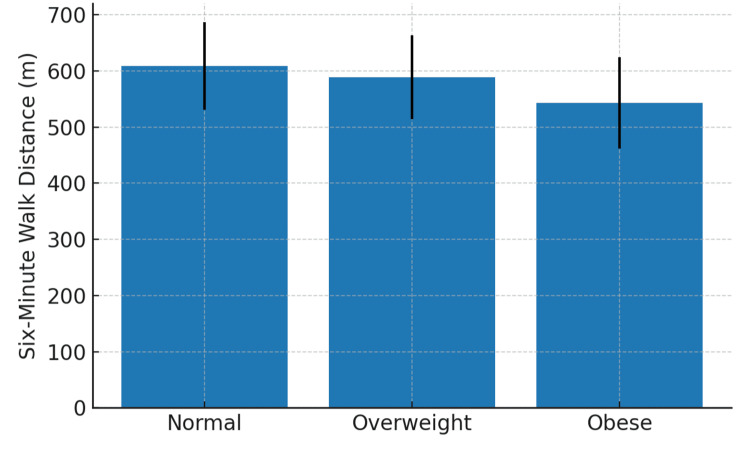
Bar graph showing mean six-minute walk distance (6MWD) in the three BMI categories.

The mean 6MWD across normal, overweight, and obese BMI groups decreases as BMI increases, with the obese group covering the shortest distance (Figure 1). 

**Figure 2:**
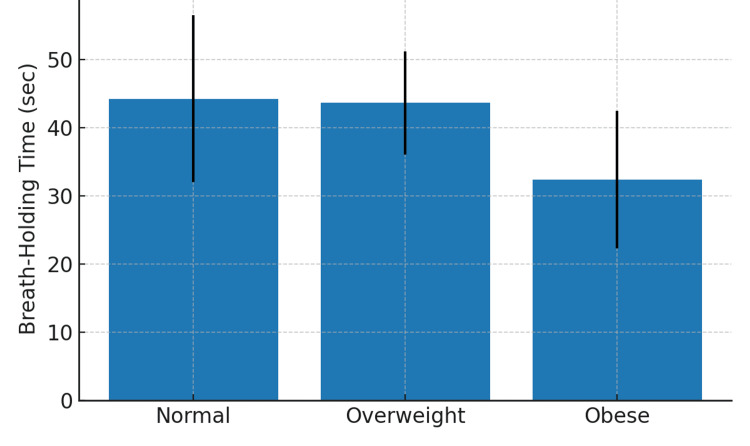
Bar graph showing mean breath-holding time (BHT) in the three BMI categories.

The mean BHT across normal, overweight, and obese BMI groups showed a similar trend of decreasing BHT with higher BMI (Figure 2).

## Discussion

The present study demonstrates that higher BMI, particularly obesity, is associated with a significant reduction in BHT and a non-significant trend toward reduced 6MWD in apparently healthy adults aged 20-40 years. Although the reduction in 6MWD did not achieve statistical significance, the direction of change suggests that a larger sample size - particularly within the obese category - might reveal a statistically significant association.

The inverse relationship between BMI and BHT observed in our study can be explained by the mechanical and physiological changes that excess adiposity imposes on the respiratory system. In obesity, fat accumulation occurs not only subcutaneously but also within the mediastinum and abdominal cavities. These deposits can alter the mechanical properties of both the lungs and the chest wall, thereby reducing their compliance. Reduced compliance increases the work of breathing, limits lung expansion, and may shorten the duration an individual can voluntarily hold their breath. These findings are in agreement with earlier literature, which has documented that obesity impairs respiratory mechanics and reduces respiratory muscle efficiency [11].

Similarly, the observed decline in 6MWD with increasing BMI aligns with previous studies that have shown impaired submaximal exercise capacity in overweight and obese individuals [12]. Even though the difference in 6MWD between groups did not reach statistical significance in this study, the pattern suggests a physiologically relevant trend. It is plausible that the lack of significance is attributable to the relatively small number of participants in the obese category. A larger and more balanced sample distribution could potentially detect a meaningful difference.

Several mechanisms may explain the reduced respiratory performance in individuals with higher BMI. Obesity has been associated with decreased functional residual capacity (FRC) and expiratory reserve volume (ERV), both of which limit the volume of air available for gas exchange during exertion. Additionally, excess body mass increases the metabolic cost of physical activity, thereby placing greater demand on both the respiratory and cardiovascular systems. These combined effects can contribute to earlier onset of breathlessness and reduced endurance in activities such as the 6MWT [3,13].

From a clinical perspective, our findings reinforce the importance of early lifestyle interventions in overweight and obese individuals, even in those who are young and otherwise healthy. Maintaining optimal BMI may help preserve both respiratory reserve and exercise tolerance, thereby reducing long-term cardiopulmonary risk.

Study limitations

Despite its contributions, this study has several limitations. First, there was unequal distribution of participants across the groups. The obese group was relatively low compared to the other BMI categories, which may have limited the statistical power to detect significant differences, particularly in 6MWD. Second, as a cross-sectional study, our findings demonstrate associations but cannot establish causal relationships between BMI and respiratory performance. Third, spirometry, plethysmography and body composition analysis were not performed, which could have provided more detailed insights into the respiratory mechanics and fat distribution patterns influencing performance outcomes. 

Future directions

Future research should aim to recruit larger numbers of obese participants to better assess the magnitude of differences in BHT and 6MWD. Incorporating detailed pulmonary function testing, such as spirometry and lung volume measurements, along with body composition analysis, could provide a more comprehensive understanding of the mechanisms linking excess adiposity to respiratory performance. Longitudinal studies would also be valuable in exploring the temporal relationship between increasing BMI and decline in pulmonary and functional capacity.

## Conclusions

Obesity is associated with reduced breath-holding time and a tendency towards lower 6MWD in healthy young adults. These functional impairments may precede overt respiratory disease and highlight the importance of weight management for maintaining respiratory and exercise capacity. Early screening for subtle respiratory and functional limitations in overweight individuals could aid in timely intervention. Further longitudinal studies are warranted to explore whether these early deficits predict future respiratory morbidity.
